# Effects of Shape and Size of Cobalt Phosphate Nanoparticles against *Acanthamoeba castellanii*

**DOI:** 10.3390/pathogens8040260

**Published:** 2019-11-22

**Authors:** Ayaz Anwar, Leong Chi Fung, Areeba Anwar, Priyanka Jagadish, Arshid Numan, Mohammad Khalid, Syed Shahabuddin, Ruqaiyyah Siddiqui, Naveed Ahmed Khan

**Affiliations:** 1 Department of Biological Sciences, School of Science and Technology, Sunway University, Subang Jaya 47500, Selangor, Malaysia; chi.l22@imail.sunway.edu.my (L.C.F.);; 2 Graphene and Advanced 2D Materials Research Group, School of Science and Technology, Sunway University, Subang Jaya 47500, Selangor, Malaysia; priyankaj@sunway.edu.my (P.J.); numan.arshed@gmail.com (A.N.); khalids@sunway.edu.my (M.K.); 3 State Key Laboratory of ASIC and System, SIST, Fudan University, Shanghai 200433, China; 4 Research Centre for Nano-Materials and Energy Technology, School of Science and Technology, Sunway University, Subang Jaya 47500, Selangor, Malaysia; syeds@sunway.edu.my; 5 Department of Biology, Chemistry and Environmental Sciences, College of Arts and Sciences, American University of Sharjah, University City, Sharjah 26666, UAE; ruqaiyyah_s@hotmail.com

**Keywords:** cobalt phosphate nanoparticles, size selectivity, antiamoebic, *Acanthamoeba*

## Abstract

T4 genotype *Acanthamoeba* are opportunistic pathogens that cause two types of infections, including vision-threatening *Acanthamoeba* keratitis (AK) and a fatal brain infection known as granulomatous amoebic encephalitis (GAE). Due to the existence of ineffective treatments against *Acanthamoeba*, it has become a potential threat to all contact lens users and immunocompromised patients. Metal nanoparticles have been proven to have various antimicrobial properties against bacteria, fungi, and parasites. Previously, different types of cobalt nanoparticles showed some promise as anti-acanthamoebic agents. In this study, the objectives were to synthesize and characterize the size, morphology, and crystalline structure of cobalt phosphate nanoparticles, as well as to determine the effects of different sizes of cobalt metal-based nanoparticles against *A. castellanii*. Cobalt phosphate octahydrate (CHP), Co_3_(PO_4_)_2_•8H_2_O, was synthesized by ultrasonication using a horn sonicator, then three different sizes of cobalt phosphates Co_3_(PO_4_)_2_ were produced through calcination of Co_3_(PO_4_)_2_•8H_2_O at 200 °C, 400 °C and 600 °C (CP2, CP4, CP6). These three types of cobalt phosphate nanoparticles were characterized using a field emission scanning electron microscope (FESEM), energy dispersive X-ray spectroscopy (EDX), and X-ray diffraction (XRD) analysis. Next, the synthesized nanoparticles were subjected to biological assays to investigate their amoebicidal, amoebistatic, anti-encystation, and anti-excystation effects against *A. castellanii*, as well as cell cytotoxicity. The overall results showed that 1.30 ± 0.70 µm of CHP microflakes demonstrated the best anti-acanthemoebic effects at 100 µg/mL, followed by 612.50 ± 165.94 nm large CP6 nanograins. However, amongst the three tested cobalt phosphates, Co_3_(PO_4_)_2_, the smaller nanoparticles had stronger antiamoebic effects against *A. castellanii*. During cell cytotoxicity analysis, CHP exhibited only 15% cytotoxicity against HeLa cells, whereas CP6 caused 46% (the highest) cell cytotoxicity at the highest concentration, respectively. Moreover, the composition and morphology of nanoparticles is suggested to be important in determining their anti-acathamoebic effects. However, the molecular mechanisms of cobalt phosphate nanoparticles are still unidentified. Nevertheless, the results suggested that cobalt phosphate nanoparticles hold potential for development of nanodrugs against *Acanthamoeba*.

## 1. Introduction

*Acanthamoeba* is an opportunistic protist that is ubiquitous in nature [1]. Trophozoite and cyst are two distinctive forms of *Acanthamoeba* in its life cycle. Trophozoites are metabolically active cells that feed on other microorganisms to grow and reproduce via binary fission; cysts are dormant cells that form double-walled cell membranes to ensure their survivability under harsh conditions [2]. The uniqueness of trophozoites includes spiny acanthopodia on their cell membrane for surface attachment, movement, and predation, while cysts are able to survive for several years under harsh conditions [3,4]. Based on the differences in the nuclear small ribosomal subunit gene sequences (18S rDNA) of *Acanthamoeba*, at least 21 genotypes of *Acanthamoeba* have been identified, known as T1 to T21 [5,6]. The T4 genotype is potentially pathogenic to humans and is the major cause of two main infections, namely, granulomatous amoebic encephalitis (GAE) and *Acanthamoebic* keratitis (AK) [7].

*Acanthamoeba* is an opportunistic pathogen, which causes the central nervous system infection called GAE. This infection is rare, but its mortality rate in immunocompromised patients is high [8,9,10,11]. By 2004, approximately 150 GAE cases had been reported globally, but no more than 10 patients survived from this infection [12]. AK is a corneal disease that has detrimental effects on the human eye and can cause blindness [13]. AK is also easily misdiagnosed as other types of keratitis and is therefore often treated with incorrect, ineffective drugs [14,15]. Its reported morbidity rate was 0.01–1.49 per 10,000 asymptomatic contact lens users [16]. Currently, a combination of drugs including biguanide, amidine, and azole are prescribed for *Acanthamoeba* infections, but patients require a few months to extirpate the toxic effects of this mixture of chemo drugs [7,17,18].

The applications of nanotechnology can be categorized into disease diagnosis, target-specific drug delivery systems, and molecular imaging. Metal nanoparticles are one of the significant representatives of a target-specific drug carrier. This is because they contain unique characteristics, where they can be designed and molded with a variety of chemical functional groups to enable the conjugation of specific ligands or drugs of interest [19]. Moreover, metal nanoparticles have helped to fix the common issue of most oral drugs, where their effects are impracticable in vivo because they are easily degraded by endogenous enzymes in the gastrointestinal (GI) tract [20]. Currently, common metal nanoparticles applied in biological study are gold, platinum, silver, titanium, zinc, cerium, iron, and thallium [21]. Other than metal nanoparticles, the oxides, hydroxides, sulphides, phosphates, fluorides, and chlorides of metals have been intensively investigated for use in the biomedical field [21]. Recently, our team has published a few articles based on the application of nanodrugs against *A. castellanii*. For instance, two studies were performed to test the effects of gold nanoparticle-conjugated chlorhexidine and cinnamic acid as anti-acanthamoebic agents [22,23]. Silver nanoparticles were used to conjugate with antifungal drugs, which include amphotericin B, nystatin, and fluconazole [24]. From the studies where metal nanoparticles served as drug carriers, most of the nanodrugs exhibited stronger anti-acanthamoebic properties as compared to the drugs alone. Although studies of nanoparticle-conjugated drugs against microbial infections are well known and a trending type of research, studies on the anti-acanthamoebic effects of metal nanoparticles alone are rare [25,26].

Cobalt is an important constituent of the human body and is present in the essential vitamin B_12_ in the form of cobalamin, which serves as a cofactor for DNA synthesis and the formation of erythrocytes in the human nervous system. It is also involved in the metabolism of both fatty acids and amino acids in neurons [27]. Previous reports have shown that cellulose–cobalt and cobalt oxide nanoparticles exhibit antibacterial properties against opportunistic pathogens including Gram-positive and Gram-negative bacteria [28,29]. Another study has also proven that cobalt nanoparticles are effective antifungal agents against *Candida* species [30]. Moreover, antiviral properties were also found in cobalt nanoparticles, as they effectively kill viruses such as vesicular stomatitis virus (VSV), varicella-zoster virus (VZV), and human immunodeficiency virus type 1 (HIV-1) [31]. On the other hand, cobalt nanoparticles were also tested against malaria and dengue vectors. This study showed the antiparasitic effect of cobalt nanoparticles to *Aedes aegypti* [32]. The cobalt (II)–lapachol complex has also been proven to have anti-acanthamoebic activity [33]. Previously, the anti-acanthamoebic effects of three types of cobalt nanoparticles, which include cobalt oxide nanograins (Co_3_O_4_), cobalt phosphate microflakes (Co_3_(PO_4_)_2_), and cobalt hydroxide nanoflakes (Co_3_(OH)_2_), were studied, and cobalt phosphate microflakes were found to be the most effective anti-acanthamoebic agent [34]. 

In the present investigation, cobalt phosphate nanoparticles with different particle sizes were synthesized to identify the effect of nanoparticle size on the anti-acanthamoebic efficacies on both forms of *A. castellanii*. For this purpose, four kinds of cobalt phosphate nanoparticles including cobalt phosphate octahydrate, Co_3_(PO_4_)_2_•8H_2_O, and 3 different sizes of cobalt phosphates, Co_3_(PO_4_)_2_, were synthesized and selected for this study. Synthesized nanoparticles were characterized using X-ray diffraction (XRD) and a field emission scanning electron microscope (FESEM). These nanoparticles were then subjected to amoebicidal, amoebistatic, encystation, excystation, and cell cytotoxicity assays to study their anti-acanthamoebic ability.

## 2. Results

### 2.1. X-ray Diffraction (XRD)

**Figure 1:**
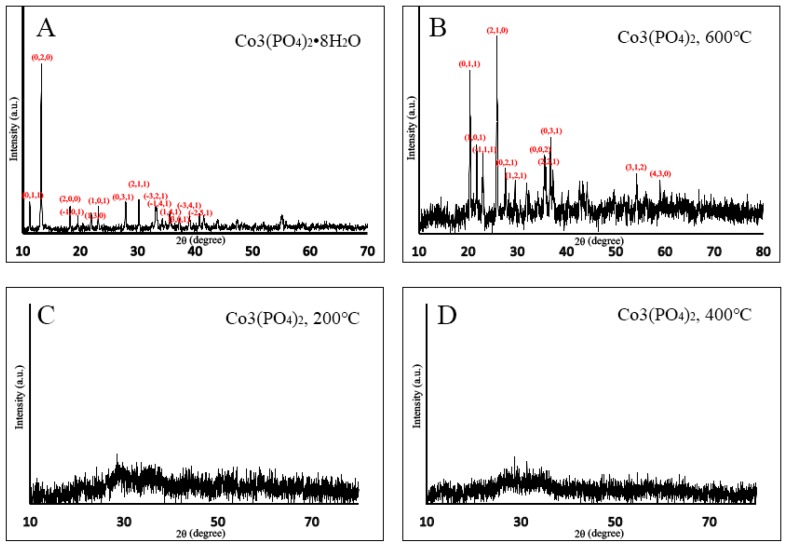
XRD patterns of (**A**) CHP; (**B**) CP6; (**C**) CP2; (**D**) CP4.

In Figure 1a, the XRD pattern of CHP exhibited dominant peaks at 2θ with the values of 11.26°, 13.27°, 18.28°, 19.62°, 21.97°, 23.19°, 27.97°, 30.31°, 33.18°, 33.37°, 35.67°, 37.31°, 40.79°, and 41.53° that correspond to the lattice planes of (1,1,0), (0,2,0), (2,0,0), (−1,0,1), (1,3,0), (1,0,1), (0,3,1), (2,1,1), (−3,2,1), (−1,4,1), (1,4,1), (3,0,1), (−3,4,1), and (−2,5,1), respectively. All dominant peaks of CHP were well indexed with their structure (PDF 00-033-0432, space group P-3m1). Figure 1b shows that CP6 displayed sharp diffraction peaks at 2θ values of 20.49°, 21.82°, 23.02°, 25.88°, 27.68°, 30.63°, 35.50°, 36.79°, 37.51°, 54.34°, and 59.10° in accordance with the lattice planes of (0,1,1), (1,0,1), (−1,1,1), (2,1,0), (0,2,1), (1,2,1), (0,0,2), (2,2,1), (0,3,1), (3,1,2), and (4,3,0), respectively. These peaks were indexed to the structure of CP6 (PDF 00-013-0503, space group P-3m1). For both cobalt nanoparticles of CP2 and CP4 in Figure 1c,d, the sharp and dominant diffraction peaks were unidentified due to their amorphous nature. 

### 2.2. Field Emission Scanning Electron Microscopy (FESEM) and Energy Dispersive X-ray (EDX)

**Figure 2:**
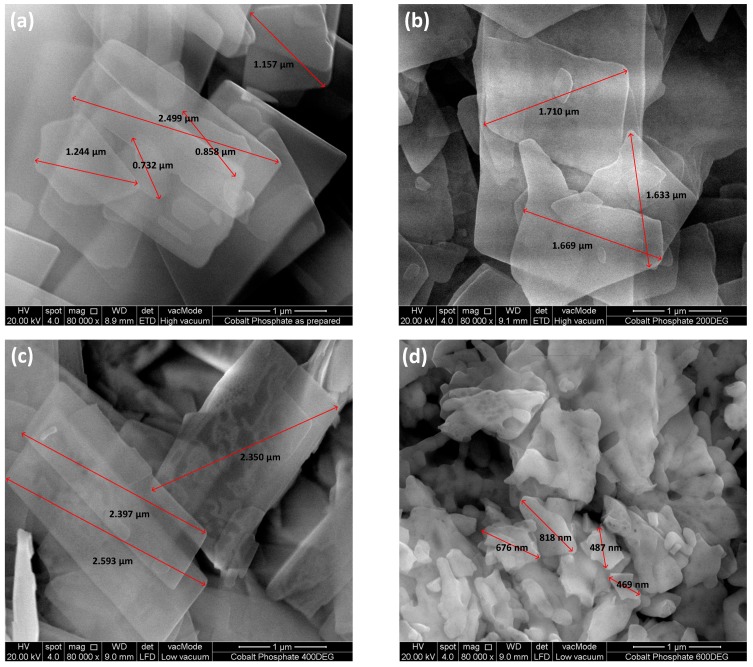
Field emission scanning electron microscope (FESEM) images of cobalt phosphate samples with a scale bar of 1 µm: (**a**) CHP; (**b**) CP2; (**c**) CP4; and (**d**) CP6.

FESEM analysis revealed that CHP exhibited a flake like morphology in the micro range with a size of 1.30 ± 0.70 µm (Figure 2). When CHP particles were calcined at 200 °C and 400 °C, a grain growth phenomenon was observed. Therefore, the microflake size of both CP2 and CP4 was increased to 1.67 ± 0.04 µm and 2.45 ± 0.13 µm, respectively. At a calcination temperature of 600 °C, the microflakes were broken down into smaller nanograins, and the average size obtained from the FESEM analysis for CP6 was found to be 612.50 ± 165.94 nm (Figure 2). According to EDX, the oxygen percentage was found to be decreasing as a trendline when the temperature was increased in the calcination process. The results showed that CHP (as prepared) contained the highest percentage of oxygen content followed by CP2, CP4, and CP6, at 78.26%, 73.05%, 69.76%, and 68.99%, respectively (Table 1). 

### 2.3. Cobalt nanoparticles Exhibited Amoebicidal Effects Against A. castellanii

**Figure 3:**
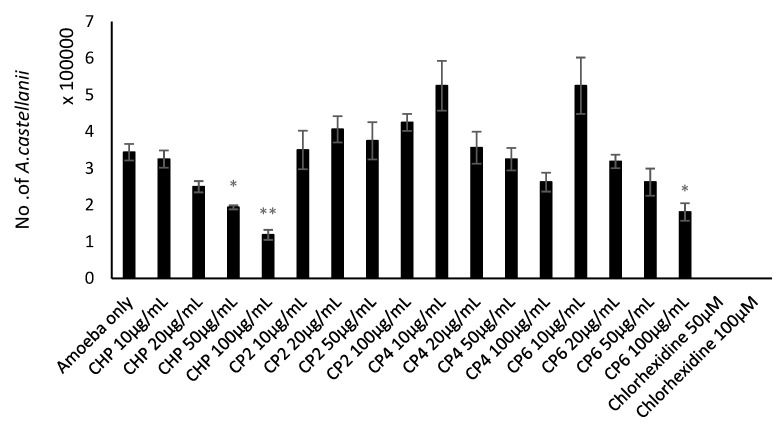
The amoebicidal activity of different sizes of cobalt nanoparticles (CHP, CP2, CP4, and CP6) against *A. castellanii* at concentrations of 10, 20, 50, and 100 µg/mL, at 24 h, 30 °C. *A. castellanii* trophozoites were incubated with chlorhexidine (positive control) and amoeba alone (negative control). After 24 h, the number of viable trophozoites was enumerated by Trypan blue exclusion assay. The results obtained represent the mean and standard error of three independent reproducible experiments performed in duplicate. *, *P* < 0.05 and **, *P* < 0.01, using a two-sample t-test and two-tailed distribution.

**Figure 4:**
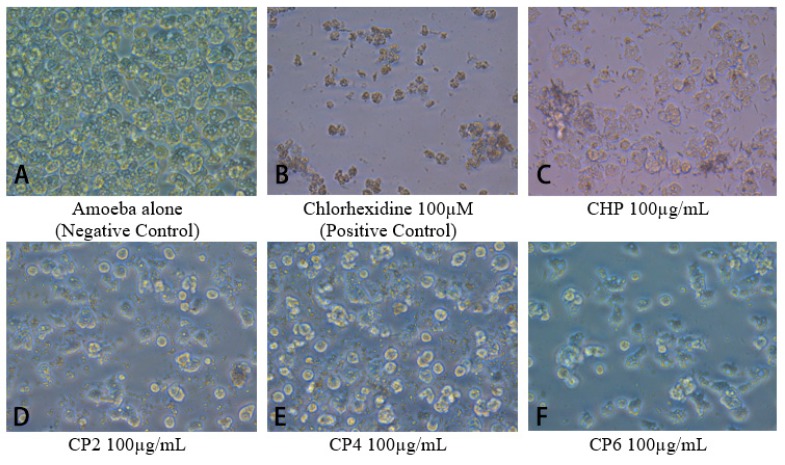
Images of different cobalt nanoparticles tested against *A. castellanii* at 100 µg/mL were captured by a phase contrast inverted microscope at 200× magnification.

Amoebicidal assays were performed to determine the effects of different sizes of cobalt nanoparticles on viability of *A. castellanii*. The number of viable *A. castellanii* was reduced to 3.4 × 10^5^ following 24 h incubation in the negative control. The results also show that CHP, CP4, and CP6 decreased the viability of *A. castellanii* in a concentration-dependent manner. However, only CHP exhibited statistically significant amoebicidal effects as compared to other nanoparticles tested. For CHP at 100 µg/mL, the number of *A. castellanii* decreased to 1.19 × 10^5^ (from 5 × 10^5^), while CP6 significantly reduced the number of viable *A. castellanii* to 1.81 × 10^5^ (from 3.4 × 10^5^) at 100 µg/mL (*P* < 0.05 using the two-sample t-test and two-tailed distribution) (Figure 3). In short, CHP displayed the strongest amoebicidal ability of 65%, followed by CP6, which reduced the number of viable *A. castellanii* at 100 µg/mL concentration to (47% of Figure 3). Representative images of *A. castllanii* before and after treatment with CoNPs are depicted in Figure 4.

### 2.4. Cobalt nanoparticles Inhibited Growth of A. castellanii

**Figure 5:**
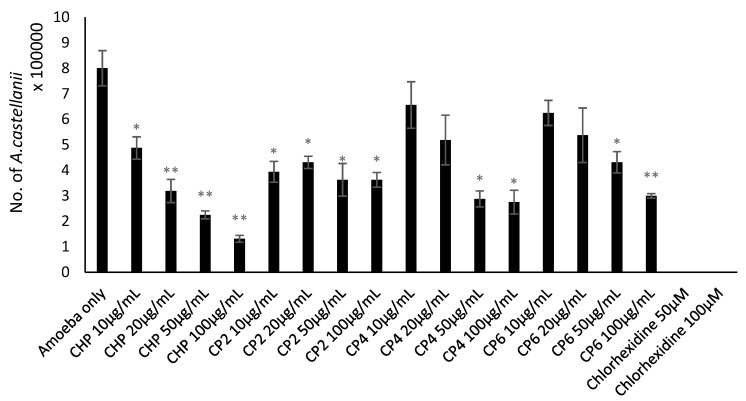
The amoebistatic activity of different sizes of cobalt nanoparticles (CHP, CP2, CP4, and CP6) against *A. castellanii* at concentrations of 10, 20, 50, and 100 µg/mL, after 24 h, at 30 °C. *A. castellanii* trophozoites were incubated with chlorhexidine (positive control) and amoeba alone (negative control). After 24 h, the number of viable trophozoites was enumerated by Trypan blue exclusion assay. The results obtained represent the mean and standard error of the independent reproducible experiments performed in duplicate. *, *P* < 0.05 and **, *P* < 0.01, using a two-sample t-test and two-tailed distribution.

**Figure 6:**
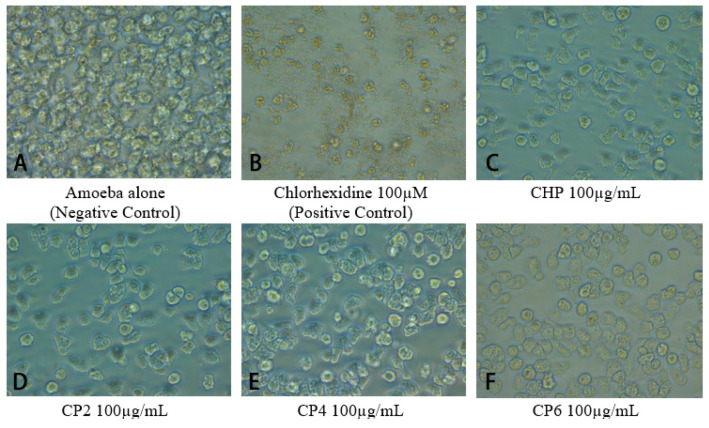
Microscopic images of *A. castellanii* treated with cobalt nanoparticles at 100 µg/mL. The images were recorded at 200x magnification.

Growth inhibition assays were conducted to determine the amoebistatic effects of the different sizes of cobalt nanoparticles against *A. castellanii*. In the negative control, the number of *A. castellanii* increased to 8 × 10^5^ following 24 h incubation (Figure 5). The results show that growth inhibitory effects were exhibited by all cobalt nanoparticles. CHP exhibited significant amoebistatic effects at all concentrations where the number of *A. castellanii* was lowered to 1.3 × 10^5^ (from 8 × 10^5^) at 100 µg/mL, whereas CP4 at 100 µg/mL also revealed a significant decrease in trophozoite cell growth where the number of *A. castellanii* was lowered to 2.75 × 10^5^ (from 8 × 10^5^) at the highest concentration. In addition, CP6 displayed significant growth inhibition effects at 20 µg/mL onwards, where the number of *A. castellanii* was reduced to 3.0 × 10^5^ (from 8 × 10^5^) at 100 µg/mL. Moreover, CP2 exhibited growth inhibition effects against *A. castellanii* in a concentration-dependent manner, of up to 45% (100 µg/mL) of cell growth compared to 80% in the negative control (amoeba alone) (Figure 5). In particular, CHP expressed the strongest amoebistatic activity, which inhibited up to 84% of trophozoites’ cell growth. Figure 6 depicts images of each well from the amoebistatic assay displaying the growth inhibition effects.

### 2.5. Cobalt nanoparticles Suppressed Encystation Effects of A. castellanii

**Figure 7:**
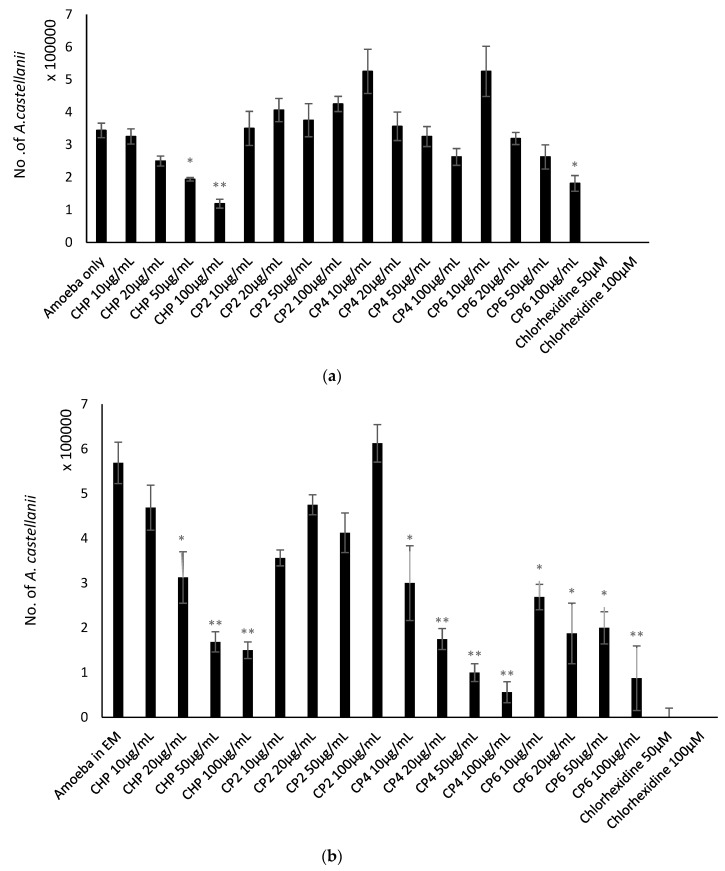
(**a**) Effects of different sizes of cobalt nanoparticles (CHP, CP2, CP4, and CP6) on encystation of *A. castellanii* at concentrations of 10, 20, 50, and 100 µg/mL after 72 h at 30 °C. *A. castellanii* trophozoites were incubated with chlorhexidine (positive control) and amoeba alone in phosphate-buffered saline (PBS) with encystation medium (EM), 50 mM MgCl_2_, and 10% glucose) as a negative control. After 72 h, the number of mature cysts were counted using a hemocytometer, and 0.1% sodium dodecyl sulphate (SDS) was used to dissolve remaining trophozoites. The results obtained represent the mean and standard error of three independent reproducible experiments performed in duplicate. * *P* < 0.05, ** *P* < 0.01 and *** *P* < 0.001, using a two-sample t-test and two-tailed distribution. (**b**) The effects of different sizes of cobalt nanoparticles (CHP, CP2, CP4, and CP6) on excystation of *A. castellanii* at concentrations of 10, 20, 50, and 100 µg/mL at 72 h and 30 °C. *A. castellanii* cysts were incubated with chlorhexidine (positive control) and amoeba alone (negative control). After 72 h, the number of viable trophozoites was enumerated by Trypan blue exclusion assay. The results obtained represent the mean and standard error of three independent reproducible experiments performed in duplicate. * *P* < 0.05, ** *P* < 0.01 and *** *P* <0.001, using a two-sample t-test and two-tailed distribution.

Encystation assays were carried out to determine the effects of different sizes of cobalt nanoparticles on the ability of *A. castellanii* to form cysts. Following 72 h incubation, 5.7 × 10^5^ cysts were formed in negative control. CHP exhibited anti-encystation effects at 100 µg/mL, where the number of the cysts was reduced to 1.5 × 10^5^ (from 5.7 × 10^5^ cysts); CP4 depicted significant anti-encystation effects at four different concentrations, and only 11.3% of cysts were left (from 5.7 × 10^5^ cysts) at 100 µg/mL. Moreover, CP6 displayed significant cyst-transforming suppression properties at all concentrations, and only 0.87 × 10^5^ of cysts were enumerated at 100 µg/mL (from 5.7 × 10^5^ cysts) (*P* < 0.05 using a two-sample t-test and two-tailed distribution) (Figure 7a). In brief, CP4 was the most potent anti-encystation agent among the four cobalt nanoparticles, as it suppressed up to 90% of trophozoites from forming into cysts. 

### 2.6. Cobalt nanoparticles Inhibited Emergence of Trophozoites from Cysts

The objective of this assay was to study the effects of different sizes of cobalt nanoparticles on the ability of *A. castellanii* to re-emerge from the cyst stage as trophozoites. According to the results, the negative control revealed the emergence of up to 2.88 × 10^5^ *A. castellanii* trophozoites after 72 h incubation. However, all four types of cobalt nanoparticles displayed potent trophozoite-transforming suppression effects. CHP and CP6 exhibited 100% anti-excystation effects at 100 µg/mL, while CP2 and CP4 also delivered strong and significant excystation inhibition properties and the total number of trophozoites enumerated were 0.125 × 10^5^ (from 2.88 × 10^5^ cysts), (*P* < 0.05 using a two-sample t-test and two-tailed distribution) (Figure 7b). In short, the four different cobalt nanoparticles exhibited potent anti-excystation effects against *A. castellanii* cysts at higher concentrations.

### 2.7. Cobalt nanoparticles Exhibited Low to Moderate Cytotoxic Effects against HeLa Cells

**Figure 8:**
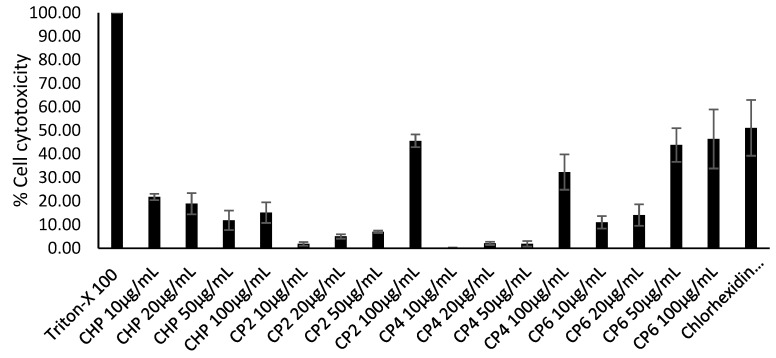
The cell cytotoxicity of various sizes of cobalt nanoparticles, which includes cobalt phosphate octahydrate (CHP) and cobalt phosphates calcinated at 200 °C, 400 °C, and 600 °C (CP2, CP4, and CP6) against HeLa cells at concentrations of 10, 20, 50, and 100 µg/mL after 24 h at 30 °C. 1% Triton-X 100 served as a positive control, while untreated HeLa cells were used as negative control. Lactate dehydrogenase (LDH) assay kit (Invitrogen) was used as per the manufacturer’s instructions.

In this experiment, the cytotoxicity of different sizes of cobalt nanoparticles against HeLa cells was evaluated. CHP demonstrated minimal toxic effects, with as low as 15% of cell cytotoxicity inhibition observed, while CP6 contributed the highest cytotoxicity against HeLa cells, showing 46% at the highest concentration, (Figure 8). Both CP2 and CP4 exhibited moderate toxic effects at 45% and 32% at 100 µg/mL, respectively.

## 3. Discussion

Nanomaterials are potential anti-acanthamoebic agents as they can serve as drug carriers as well as pathogen-targeting drugs. More importantly, they are appropriate in targeting brain infections such as GAE, as they can pass through the blood–brain barrier (BBB) [35]. Cobalt metal and its complexes are known to possess versatile antimicrobial properties, but prior to this study, the anti-acanthamoebic effects of cobalt nanoparticles had not been evaluated. 

Based on characterization of cobalt phosphate nanoparticles, XRD proved that CHP and CP6 were crystalline, whereas the structure of CP2 and CP4 was amorphous (Figure 1). Owing to the evaporation of water molecules and also grain nucleation as a result of increased thermal energy supplied during the calcination process, the CHP particles transition from a crystalline to amorphous nature for CP2 and CP4 particles. After the evaporation of the hydrate compound, the cobalt, phosphorous, and oxygen atoms re-arrange and nucleate to a more ordered crystalline domain, hence depicting a crystalline structure at 600 °C, as shown in Figure 1b. CHP as prepared was in a microsize crystalline structure; it then turned into a nanosized crystalline form when calcined at 600 °C. Therefore, 600 °C is the specific temperature that is able to break down Co_3_(PO_4_)_2_•8H_2_O (CHP) microflakes into Co_3_(PO_4_)_2_ (CP6) nanograins, and both CP2 and CP4 are the intermediates in this reaction. The EDX result also supported to statement mentioned above as the percentage of oxygen decreasing trendline was observed from CHP to CP6. This circumstance indicated that a stronger dehydration process occurred in CHP at a higher calcination temperature.

In biological studies, the crystalline structure, composition, morphology, and size of each cobalt nanoparticles were evaluated with respect to their anti-acanthamoebic effects. Amoebicidal assays (Figure 3) revealed that only the CHP and CP6 showed significant effects against *A. castellanii*. CP2 and CP4 with a flaky morphology did not significantly inhibit the viability of *Acanthamoeba* as compared to the granular shaped CP6, and this suggests that flaky morphology may be a contributory factor; however, this needs to be studied further. All cobalt nanoparticles exhibited a significant amoebistatic effect (Figure 5); however, CHP displayed the greatest growth inhibition effects against *A. castellanii*. On the other hand, all cobalt nanoparticles provided significant anti-encystation (Figure 7a) and anti-excystation (Figure 7b) properties, except CP2, which showed limited effects. Therefore, these cobalt nanoparticles except CP2 are potential novel drugs against *A. castellanii*, especially in treating GAE infection, as they can be delivered to the brain cells via the blood–brain barrier. In short, CHP has the strongest anti-acanthamoebic effects, followed by CP6; overall, CP2 and CP4 did not show effective anti-acanthamoebic effects against *A. castellanii*.

Based on the literature review, the antibacterial mechanism of cobalt nanoparticles is related to oxidative stress. Cobalt nanoparticles induced the production of reactive oxygen species (ROS), which can diffuse into bacteria through the cell membrane, causing DNA damage to bacteria [36]. This might explain the reason that CHP exhibited the most potent anti-acanthamoebic effects against *A. castellanii,* as CHP contained the highest oxygen percentage content, thereby inducing harsher oxidation stress. Another study also proposed the importance of the size of metal nanoparticles, revealing that smaller size particles contain a greater surface area to volume ratio. Thus, they are able to interact with the bacterial surface more effectively and efficiently, hence displaying greater antimicrobial properties [37]. CP6 was the second strongest anti-acanthamoebic nanoparticle. This could correlate to the small particle size of CP6, and its anti-acanthamoebic activity may have been enhanced due to effective interaction with *A. castellanii* surface due to a greater surface area to volume ratio. 

Based on results by Kedziora et al. [38], the antibacterial activity of titanium dioxide doped with silver nanoparticles in a crystalline state are more powerful than that of the amorphous form. This could be related to defects found on the surface of CP2 and CP4 nanoparticles. Based on the FESEM images, porous structures were observed on the surface, which were caused by a grain growth process during calcination. Moreover, the grain growth of cobalt nanoparticles caused agglomeration to occur, increasing the surface tension of each particle, thereby reducing the conserved reactivity of the particles for longer duration. In short, defects on the nanoparticle surface and grain growth process observed in CP2 and CP4 caused their interactions with *A. castellanii* to intensely decrease, thus reducing their anti-acanthamoebic activity. These findings are also supported by other reports [39]. In another study, it was reported that the mode of action of cobalt (II) complexes where cobalt (II) affected the electron flow in the entire complexes enables them to easily penetrate into fungal cells [40]. However, these are predicted reasons, and the actual mechanisms of anti-acanthamoebic effects of cobalt nanoparticles need to be determined and studied in detail both in vitro and in vivo.

## 4. Materials and Methods

### 4.1. Chemicals

Cobalt chloride hexahydrate (CoCl_2_.6H_2_O), di-sodium hydrogen phosphate (Na_2_HPO_4_) anhydrous and chlorhexidine were purchased from Sigma-Aldrich (San Francisco, CA, USA). Absolute ethanol, non-nutrient agar (NA) powder, proteose peptone, yeast extract, D-glucose, phosphate-buffered saline (PBS), Roswell Park Memorial Institute (RPMI-1640) powder, sodium dodecyl sulphate (SDS) powder, and Trypan Blue powder were purchased from Thermo Fisher Scientific (Massachusetts, USA).

*Synthesis of cobalt phosphate nanoparticles, Co_3_(PO_4_)_2_*

The cobalt phosphate samples were prepared by dissolving 0.2 M of di-sodium hydrogen phosphate, Na_2_HPO_4_, and 0.1 M of cobalt chloride hexahydrate, CoCl_2_•6H_2_O, in deionized water. A total of 0.2 M of Na_2_HPO_4_ was added dropwise to 0.1 M of CoCl_2_•6H_2_O solution while subjected to ultrasonication using a horn sonicator for 40 min. After 40 min, a milky pink colloidal solution was formed. This solution was centrifuged to separate the cobalt phosphate particles and washed with deionized water three times to remove unwanted impurities and washed once with absolute ethanol as a surfactant to prevent aggregation of the particles. Then, the powder was left to dry overnight. This led to the formation of the prepared sample: cobalt phosphate octahydrate, denoted as CHP. The sample was triturated with a pestle and mortar. The CHP sample was then calcined in a muffle furnace at different temperatures of 200 °C, 400 °C, and 600 °C and is denoted as CP2, CP4, and CP6.

### 4.2. Field Emission Scanning Electron Microscopy (FESEM) and Energy Dispersive X-ray (EDX)

The synthesized powder samples were placed on sample stubs covered with a double-sided carbon tape and then loaded into the FESEM machine. The surface morphology of the synthesized samples was investigated using FESEM (FEI Quanta 400F FESEM) with a magnification of 80,000 and accelerating voltage of 20 kV. The elemental composition present in the synthesized samples was measured using EDX (Oxford-Instruments INCA 400 with X-Max Detector).

### 4.3. X-ray Diffraction (XRD)

The phase identification and crystallinity properties of the synthesized samples were obtained by X-ray powder diffraction (XRD) (Cu-Kα, Bruker D8 Advance) operating at 40 kV and 40 mA. The XRD patterns were measured over an angular range 2θ of 10–80° using a Cu Kα radiation wavelength of 1.540 Å with a step size of 0.025° and step time of 19.2 s.

### 4.4. Henrietta Lacks Cervical Adenocarcinoma (HeLa) Cells Culture

HeLa cells were cultured in RPMI-1640 media, supplemented with 10% FBS, 10% Nu-serum, 2 mM glutamine, 1 mM pyruvate, penicillin (100 units/mL), streptomycin (100 µg/mL), non-essential amino acids, and vitamins in T-75 culture flasks. Cells were kept in an incubator at 37 °C, with 5% CO_2_ and 95% humidity. Cells were detached by adding 2 mL trypsin for 10 min in the incubator after formation of monolayer cells. Cell pellets were obtained by centrifugation at 2000xg for 5 min, and they were resuspended with 30 mL of fresh supplemented RPMI-1640. A total of 200 µL of cell suspension was aliquoted into 96-well culture plate and kept in the same incubator. After 24 to 48 h of incubation, a plate containing confluent HeLa monolayers was used for cell cytotoxicity assays.

### 4.5. Acanthamoeba Cultures

*A. castellanii* (ATCC 50492, strain isolated from AK patient) belonging to the T4 genotype was cultured with 10 mL of Proteose peptone, Yeast extract and glucose (PYG) media with the composition of protease peptone 0.75% (*w*/*v*), yeast extract 0.75% (*w*/*v*), and glucose 1.5% (*w*/*v*) in T-75 culture flasks. Amoebae cultures were incubated at 30 °C and the media changing process was done routinely to maintain healthy amoebae cultures. Within 24 h, 70% confluency of amoebae culture was achieved. Amoebae were detached by placing culture flasks on ice for 10 min, followed by gentle tapping [41]. Then, the amoebae cell suspension was shifted into 50 mL conical tubes and centrifuged at 3000 g for 10 min. Amoebae pellets were resuspended with fresh media for each assay. The amoebae suspension was counted using a hemocytometer, and 5 × 10^5^ of cells were loaded into 24-well culture plates for all assays.

### 4.6. Preparation of Cysts

*A. castellanii* cyst cells were prepared by inoculating *A. castellanii* trophozoites on non-nutrient agar. First, *A. castellanii* trophozoites were detached and collected from a culture flask, and centrifugation was carried out at 3000 g for 10 min. The obtained pellet was then resuspended in PBS, and 1 mL of this cell suspension was aliquoted on each non-nutrient each agar plate. Inoculated non-nutrient agar plates were left to dry in a biosafety cabinet sealed with parafilm. Lastly, the plates were incubated at 30 °C for more than 14 days [42]. Formation of cysts was observed on a daily basis, and the resulting cysts were utilized in excystation assays.

### 4.7. Amoebicidal Assay

To determine the effects of different cobalt nanoparticles on the viability of *A. castellanii* trophozoites, amoebicidal assays were conducted. Different test concentrations of cobalt nanoparticles such as 10 µg/mL, 20 µg/mL, 50 µg/mL and 100 µg/mL, were incubated with trophozoites in 24-well culture plates (5 × 10^5^ cells/well/500 μL RPMI) at 30 °C for 24 h. *A. castellanii* with chlorhexidine served as positive control, while amoeba alone was used as a negative control. After 24 h incubation, the viability of *A. castellanii* was determined by adding 0.1% Trypan blue into each well to count the unstained viable cells using a hemocytometer [24]. The data obtained are the representative (Mean ± S.E.M) of three independent experiments performed in duplicate.

### 4.8. Growth Inhibition Assay

Four types of cobalt nanoparticles were tested against *A. castellanii* to determine their amoebistatic properties. Trophozoites of 5 × 10^5^ were inoculated per well in 24-well culture plates containing PYG growth medium and different concentrations of cobalt nanoparticles. The positive and negative controls in this assay were chlorhexidine and amoeba alone, respectively. After 24 h incubation at 30 °C, 0.1% trypan blue staining was performed, and the number of live amoeba cells was evaluated with a hemocytometer [40]. The data obtained are the representative (Mean ± S.E.M) of three independent experiments performed in duplicate.

### 4.9. Encystation Assay

To identify the effects of different cobalt nanoparticles against *A. castellanii* cysts, transformation encystation assays were performed. *A. castellanii* trophozoites of 5 × 10^5^ were seeded per well in 24-well culture plates, in PBS containing encystation media (50 mM MgCl_2_ and 10% glucose) for 72 h at 30 °C [23]. Test concentrations of 10 µg/mL, 20 µg/mL, 50 µg/mL, and 100 µg/mL for each type of cobalt nanoparticles were used to test against *A. castellanii*. Chlorhexidine was used as a positive control, whereas amoeba alone was used as a negative control. After 72 h, the number of cysts per well was counted by adding 0.1% SDS to dissolve the remaining trophozoites, and only mature cysts were enumerated with a hemocytometer.

### 4.10. Excystation Assay

For excystation assays, *A. castellanii* cysts were treated with various concentrations of cobalt nanoparticles in PYG growth medium. The purpose of this assay was to study the effects of different cobalt nanoparticles in inhibiting emergence of active trophozoites. Firstly, cysts were collected by scraping with 3 mL of PBS on non-nutrient agar. Cysts of 1 × 10^5^ per well were incubated in PYG growth medium with cobalt nanoparticles in 24-well plates for 72 h at 30 °C. The positive control for this assay was *A. castellanii* cysts incubated with chlorhexidine, while the negative control was amoeba cysts alone. After 72 h of incubation, the number of trophozoites emerged was determined using a hemocytometer with 0.1% Trypan blue [43].

### 4.11. Cytotoxicity Assay

To identify the toxic effects of cobalt nanoparticles on human cells, a uniform monolayer of HeLa cells (approx. 100,000 cells / well) was grown in RPMI-1640 medium in a 96-well plate. Afterwards, RPMI-1640 was replaced with the fresh serum-free RPMI-1640 medium and different test concentrations (10 µg/mL, 20 µg/mL, 50 µg/mL, and 100 µg/mL) of cobalt nanoparticles were added. The incubation time was 24 h at 37 °C and 5% CO_2_ in a humidified incubator. Following the incubation time, 1% Triton X-100 was added to the positive control wells and incubated under the same conditions for 30 min. Untreated HeLa cells were used as negative control. Next, the cell-free supernatant was collected from each well, and Lactate dehydrogenase (LDH) cytotoxicity detection kit (Invitrogen) was used as per the manufacturer’s instructions. Absorbance of the samples was measured at 490 nm using a plate reader. The percentage of cytotoxicity of each sample was calculated using the formula: (sample absorbance – negative control absorbance) / (positive control absorbance – negative control absorbance) × 100% [44].

## 5. Conclusions

In conclusion, different sizes of cobalt nanoparticles, especially CHP and CP6, have shown potency against *A. castellanii* and hold promise as novel drugs. CHP exhibited the best overall anti-acanthamoebic effects followed by CP6. It is proposed that the size of cobalt nanoparticles plays a pivotal role in their anti-acanthamoebic activities. However, the compositions, morphology, and crystallinity of cobalt nanoparticles were also found to be highly correlated to their anti-acanthamoebic activities. Despite the potent anti-acanthamoebic effects exhibited by cobalt nanoparticles, future studies are needed to understand the exact molecular mechanisms. Furthermore, in vivo studies on cobalt nanoparticles against *Acanthamoeba* infections are also needed before justifying the drug value of these nanomaterials.

## Figures and Tables

**Table 1:** Energy-dispersive X-ray spectroscopy (EDX) analysis of cobalt nanoparticles, providing their individual elemental percentage.

Elemental Atomic Percentage (at %)	CHP	CP2	CP4	CP6
Oxygen	78.26	73.05	69.76	68.99
Phosphorus	9.74	12.87	14.03	14.84
Cobalt	12.00	14.08	16.21	16.17
